# Schwannoma of the submandibular gland: a case report

**DOI:** 10.1186/1752-1947-8-231

**Published:** 2014-06-26

**Authors:** Gaffar Aslan, Fikret Cinar, Fatmagul Kusku Cabuk

**Affiliations:** 1 Department of Otolaryngology, Florence Nightingale Hospital, Istanbul Bilim University, Abidei Hurriyet Caddesi 290, Caglayan 34381, Istanbul, Turkey; 2 Department of Pathology, Florence Nightingale Hospital, Istanbul Bilim University, Abidei Hurriyet Caddesi 290, Caglayan 34381, Istanbul, Turkey

**Keywords:** Schwannoma, Neurilemmoma, Submandibular gland, Treatment, Benign tumor

## Abstract

**Introduction:**

Schwannoma is a benign, solitary, and slowly progressive encapsulated tumor originating from the sheath of myelinated nerve fibers. Schwannoma of the salivary gland is a particularly rare form of an extracranial neurogenic tumor. Here, we present an unusual case of a schwannoma of the submandibular gland in a 19-year-old man. Total excision of the submandibular gland resulted in complete resolution of symptoms with no cranial nerve deficits. The details of the histopathologic and immunohistochemical features are presented.

**Case presentation:**

A 19-year-old Caucasian man was admitted to our clinic with a painless mass on the right side of his neck that he had had for the past four months. A neck examination revealed a smooth-surfaced, mobile, firm, and painless mass, 6cm in its greatest diameter, on the right side of the submandibular region. Fine-needle aspiration cytology was suggestive of a submandibular gland schwannoma. After the initial evaluation, our patient was prepared for surgical evaluation and resection with a presumptive diagnosis of a neurogenic tumor of the submandibular gland. The final diagnosis of schwannoma was verified by microscopic and immunohistochemical studies. At one-year follow-up of the case, there was no evidence of recurrence.

**Conclusions:**

Schwannoma of the salivary gland is a particularly rare form of an extracranial neurogenic tumor. Our findings indicate good prognosis in an unusual case of a submandibular gland schwannoma in a 19-year-old man treated by surgical excision with no recurrence within 12 months of follow-up.

## Introduction

Schwannomas are benign, solitary and well-differentiated tumors originating from Schwann cells [1]. Nearly 45 percent of all schwannomas occur in the head and neck area, where they may originate from any of the peripheral, cranial or autonomic nerves [2]. These benign tumors occur regardless of age or sex and are painless, insidious and slow growing [3]. So, they are of long duration at the time of the presentation and rarely show a rapid course [4]. The nerve of origin is not identified in around 10 to 40 percent of schwannomas [5]. Here, we present the case of a schwannoma of the submandibular gland in a 19-year-old man.

## Case presentation

**Figure 1:**
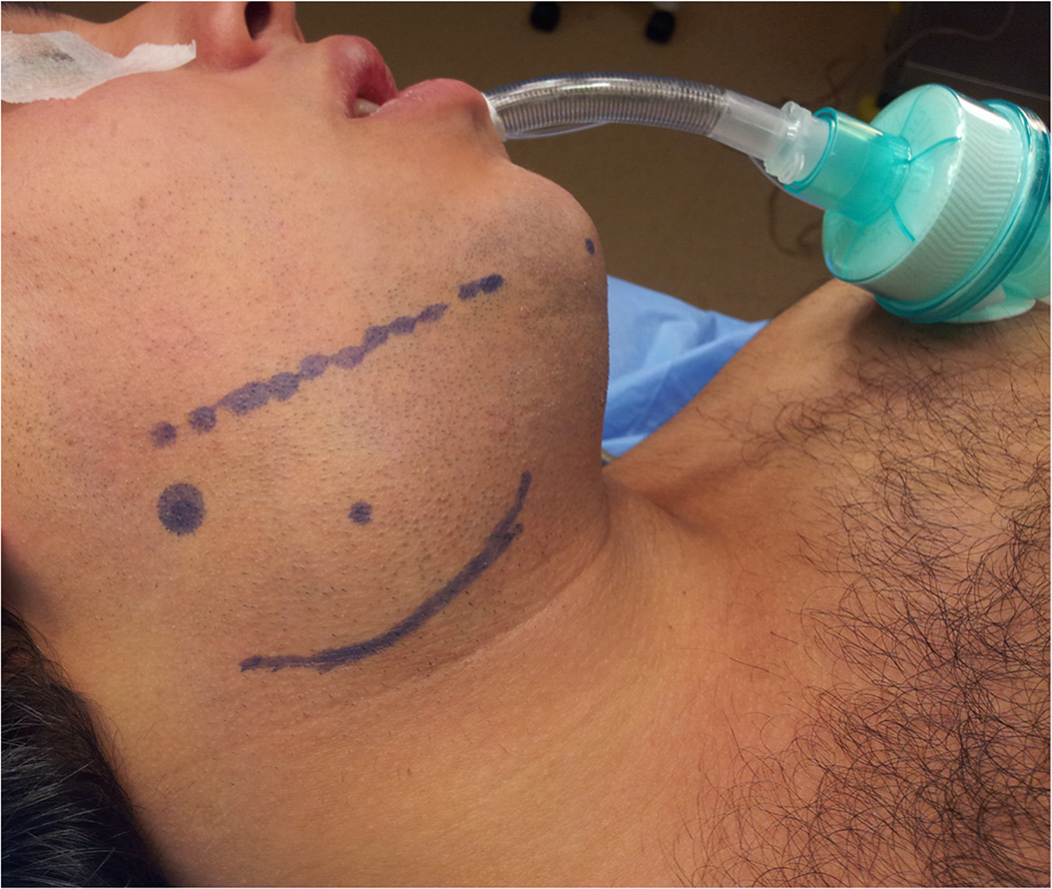
A 19-year-old man presented with a mass on his right lateral submandibular region.

**Figure 2:**
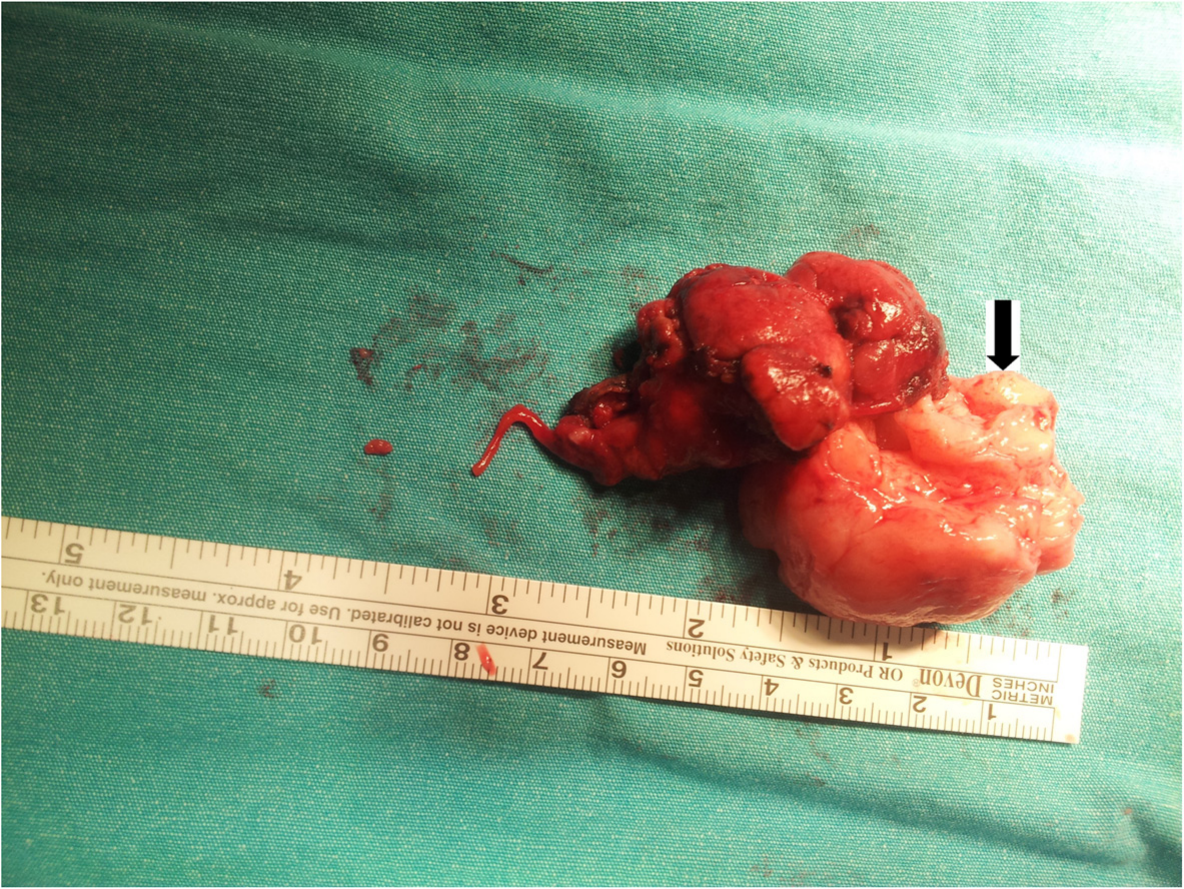
View of the tumor specimen with submandibular gland (black arrow).

**Figure 3:**
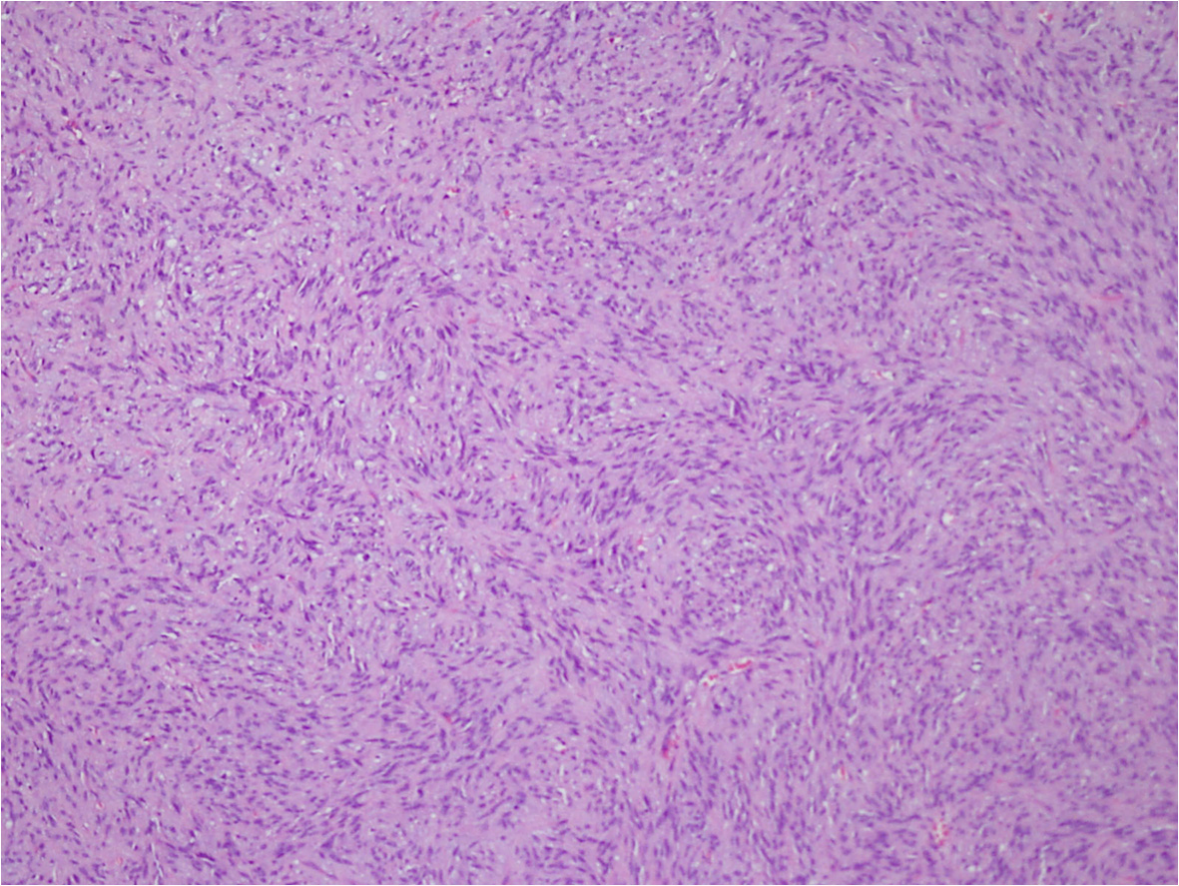
Microscopic examination revealed a well-encapsulated tumor exhibiting areas of organized spindle-shaped cells in a palisading arrangement around acellular, eosinophilic areas forming Verocay bodies giving an Antoni type A pattern (hematoxylin & eosin ×100).

**Figure 4:**
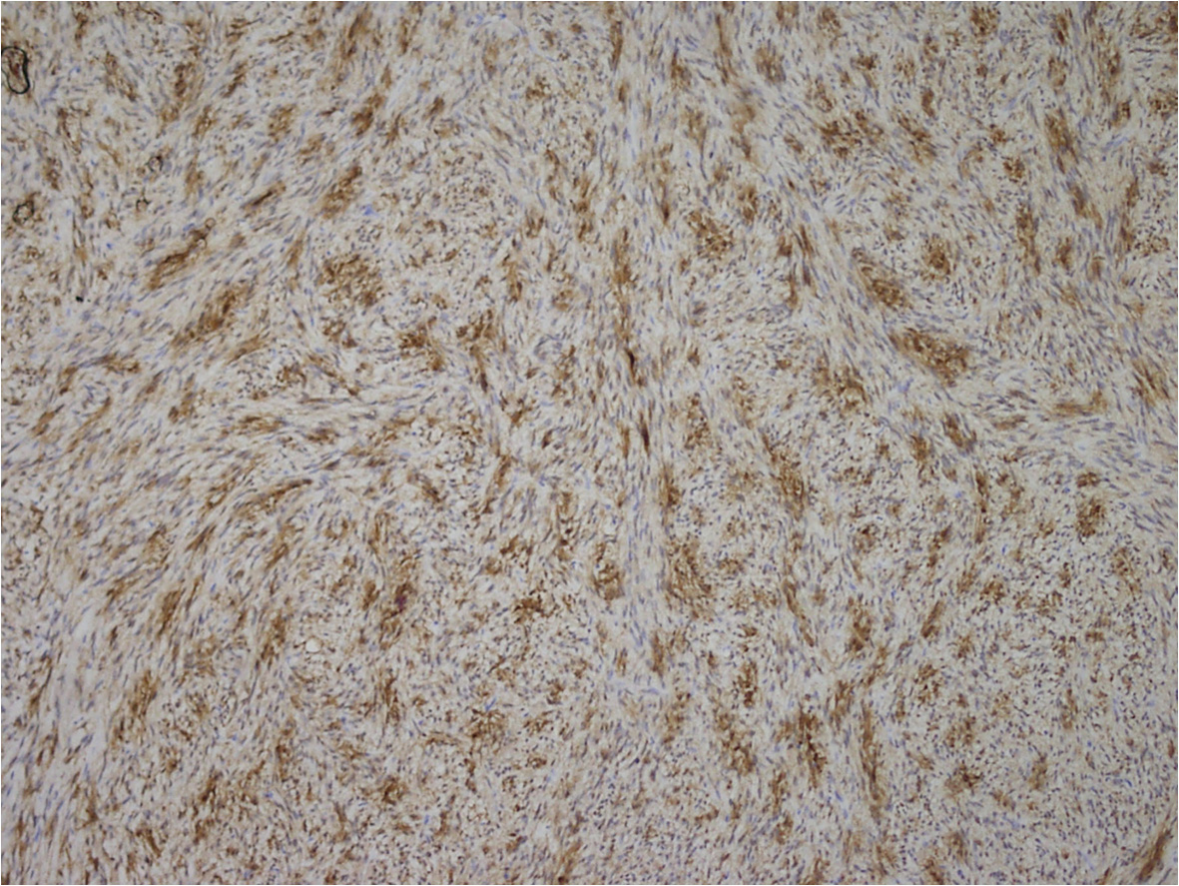
Immunohistochemical investigation of the tumor cells showed diffuse, strongly positive staining for S-100 protein (×200).

A 19-year-old Caucasian man was admitted to our clinic with a painless mass on the right side of his neck for the past four months (Figure 1). A physical examination of his neck revealed a smooth-surfaced, mobile, firm, and painless mass of 6cm in its greatest diameter, on the right side of the submandibular region. No regional lymphadenitis was detected. All the cranial nerve examinations were normal. An ultrasound examination of the neck revealed a well-circumscribed and heterogeneous mass. Fine-needle aspiration (FNA) cytology of the mass was suggestive of a submandibular gland schwannoma. After the initial evaluation, our patient was prepared for surgical evaluation and resection with a presumptive diagnosis of a neurogenic tumor of the submandibular gland.Our patient underwent surgical excision of the mass under general anesthesia. The mass was carefully dissected from the adjacent structures. The lesion was completely excised with the submandibular gland and the surgical defect was closed. Macroscopically, the resected mass was encapsulated, yellowish in color, measuring 6cm in its greatest diameter. It was oval, smooth and firm (Figure 2). Our patient had an uneventful postoperative recovery. Total excision resulted in complete resolution of symptoms with no cranial nerve deficits.A microscopic examination revealed a well-encapsulated tumor exhibiting areas of organized spindle-shaped cells in a palisading arrangement around acellular, eosinophilic areas, forming Verocay bodies giving an Antoni type A pattern (Figure 3). Atypical mitosis was not seen. Immunohistochemical investigation of the tumor cells showed diffuse, strongly positive staining for S-100 protein (Figure 4). These findings were suggestive of schwannoma.

Our patient was free of symptoms at the 12-month follow-up.

## Discussion

Schwannoma, first described in 1908 by Verocay, commonly occurs between 30 and 50 years old [6]; however, our case was a 19-year-old man. Neurilemmoma/schwannoma is a benign tumor arising from and consisting solely of Schwann cells [6]. Approximately 25 to 45 percent of extracranial schwannomas present in the head and neck area; the most commonly affected regions are the temporal bone, lateral neck, and paranasal sinuses [7]. Among the cranial nerves, schwannomas can arise from the glossopharyngeal, accessory, and hypoglossal nerves, while the most common type is acoustic neurinoma differentiating from the vestibulocochlear nerve [7]. Schwannomas are usually solitary lesions; however, some are seen as multiple lesions as part of neurofibromatosis type 1 [6].

Clinical evidence of the tumor usually does not present for a long time. The most common symptom is a slow-growing mass [1,4,7,8]. Neurological symptoms and pain are rare. Schwannomas can be seen at any age and women show dominance [8,9]. Malignant transformation is very rare [1,8-10].

Diagnostic investigations include computed tomography (CT), magnetic resonance imaging (MRI), ultrasound scan and FNA. MRI is the best choice in detecting the extent of the tumor and correlates well with the operative findings [11]. Biswas *et al.* have reported their 10 years of experience regarding extracranial head and neck schwannomas, and in their report, only 6 percent of patients could have been diagnosed preoperatively on the basis of clinical findings, CT and MRI scans, and FNA [12]. Schwannomas have specific MRI properties, including specific signs (split-fat sign, fascicular sign, target sign) and signal patterns (that is, isointense T1 signal relative to skeletal muscle; increased and slightly heterogeneous T2 signal) [13]. Diagnosis is confirmed by histopathology showing the presence of Antoni A and Antoni B cells, nuclear palisading, whirling of cells and Verocay bodies [7,12,13]. We believe this tumor originated in an autonomic nerve of the submandibular gland.

The treatment of schwannomas is problematic. Because of resistance to radiotherapy, surgical excision is necessary for optimal treatment [8,9,14,15]. Kang *et al.* reported that more than half of the surgically treated cases exhibited postoperative neural deficits that were primarily caused by iatrogenic injury to either the nerve of origin or adjacent neural ending [7]. In our case, no neural deficits or other problems presented after total excision of the tumor.

The malignant potential of extracranial schwannomas and risk of recurrence after surgical resection are unclear [14], while in most studies investigating extracranial schwannomas, recurrence or malignant transformation of the tumor have not been reported [7,12]. At one-year follow-up of the present case, there was no evidence of recurrence and prognosis was excellent. Nevertheless, while a malignant transformation of schwannoma is an exceptionally rare event, disregarding this possibility seems not to be a safe practice in light of the fact that malignancy can occur even if rarely [7,10,12,14].

## Conclusions

Schwannoma of the salivary gland is a particularly rare form of an extracranial neurogenic tumor. Our findings indicate good prognosis in an unusual case of a submandibular gland schwannoma in a 19-year-old man treated by surgical excision with no recurrence within 12 months of follow-up.

## Consent

Written informed consent was obtained from the patient for publication of this case report and any accompanying images. A copy of the written consent is available for review by the Editor-in-Chief of this journal.

## Competing interests

The authors declare that they have no competing interests.

## Authors’ contributions

GA was the primary physician of the patient, interpreted the patient data and was the major contributor to writing the manuscript. FC operated on the patient and contributed to writing the manuscript. FKC performed the histopathological examinations and contributed to writing the manuscript. All authors read and approved the final manuscript.
